# Machine Learning Methods Improve Specificity in Newborn Screening for Isovaleric Aciduria

**DOI:** 10.3390/metabo13020304

**Published:** 2023-02-18

**Authors:** Elaine Zaunseder, Ulrike Mütze, Sven F. Garbade, Saskia Haupt, Patrik Feyh, Georg F. Hoffmann, Vincent Heuveline, Stefan Kölker

**Affiliations:** 1 Engineering Mathematics and Computing Lab (EMCL), Interdisciplinary Center for Scientific Computing (IWR), Heidelberg University, 69120 Heidelberg, Germany; 2 Data Mining and Uncertainty Quantification (DMQ), Heidelberg Institute for Theoretical Studies (HITS), 69118 Heidelberg, Germany; 3 Division of Child Neurology and Metabolic Medicine, Center for Child and Adolescent Medicine, Heidelberg University Hospital, 69120 Heidelberg, Germany

**Keywords:** data analysis, artificial intelligence, data mining, isovaleric acidemia, neonatal screening, inborn error of metabolism

## Abstract

Isovaleric aciduria (IVA) is a rare disorder of leucine metabolism and part of newborn screening (NBS) programs worldwide. However, NBS for IVA is hampered by, first, the increased birth prevalence due to the identification of individuals with an attenuated disease variant (so-called “mild” IVA) and, second, an increasing number of false positive screening results due to the use of pivmecillinam contained in the medication. Recently, machine learning (ML) methods have been analyzed, analogous to new biomarkers or second-tier methods, in the context of NBS. In this study, we investigated the application of machine learning classification methods to improve IVA classification using an NBS data set containing 2,106,090 newborns screened in Heidelberg, Germany. Therefore, we propose to combine two methods, linear discriminant analysis, and ridge logistic regression as an additional step, a digital-tier, to traditional NBS. Our results show that this reduces the false positive rate by 69.9% from 103 to 31 while maintaining 100% sensitivity in cross-validation. The ML methods were able to classify mild and classic IVA from normal newborns solely based on the NBS data and revealed that besides isovalerylcarnitine (C5), the metabolite concentration of tryptophan (Trp) is important for improved classification. Overall, applying ML methods to improve the specificity of IVA could have a major impact on newborns, as it could reduce the newborns’ and families’ burden of false positives or over-treatment.

## 1. Introduction

Starting more than 50 years ago, newborn screening (NBS) programs aim at early, ideally pre-symptomatic identification of individuals with treatable severe rare diseases to reduce morbidity and mortality. They are highly successful instruments of secondary prevention with a growing panel of different conditions [1,2]. Isovaleric aciduria (IVA; OMIM #243500) is an organic aciduria leading to severe life-threatening (neonatal) metabolic compensations in its severest form. It is caused by bi-allelic pathogenic variants in the IVD gene (cytogenic location: 15q15.1), resulting in a deficiency of isovaleryl-CoA dehydrogenase (IVD, EC 1.3.99.10) in the leucine degradation pathway and hence accumulation of metabolites deriving from isovaleryl-CoA. After the introduction of tandem mass spectrometry in NBS, IVA became a target condition in German regular NBS in 2005 [3]. Identification by NBS leads to earlier specialized treatment and thus, reduced mortality for affected individuals with the classical disease course [4]. However, the inclusion of IVA into the NBS disease panel also resulted in the identification of individuals with an attenuated, possibly asymptomatic, disease variant (so-called “mild” IVA [4,5]), which was virtually unknown in the pre-screening era. By this, the estimated birth prevalence of IVA increased from 1 in 280,000 newborns to 1 in 90,000–100,000 newborns worldwide [4,6,7]. A multi-center long-term observational study showed individuals with mild IVA, 80% of the screened with IVA in Germany, to be at risk for over-treatment [4]. The second struggle of NBS for IVA is an increasing number of false positives due to the increasing use of pivmecillinam, an antibiotic, used in urinary tract infections in pregnant women [8]. The antibiotic’s metabolite pivaloylcarnitine is isobaric to isovalerylcarnitine (C5), the primary biomarker in NBS for IVA [9]. New approaches to improve IVA NBS’s specificity (reduction of false positives) while maintaining 100% sensitivity and a better distinction and prediction for the IVA disease course (mild versus classic) are urgently needed to reduce the newborns’ and families’ burden of false positives or over-treatment.

Recently, in medical applications, machine learning (ML) methods, a sub-field of artificial intelligence (AI), have been applied in various areas such as image classification for mammography interpretation [10,11], diabetes prediction [12], and lung cancer screening [13,14]. In the context of NBS, a variety of supervised ML methods have been applied to NBS data to predict whether or not a newborn suffers from a condition. The methods and their results were summarized in a recent review [15]. They enabled a reduction of false positive rates and identification of so far unknown metabolic patterns by relying on complex feature combinations instead of predefined single cut-off values. Among the previously applied ML methods, logistic regression (LR) and support vector machine (SVM) were established as valuable candidates for NBS classification and achieved high performance in comparative studies [15]. In particular, ML methods showed promising results for improving specificity for phenylketonuria [16,17,18], methylmalonic aciduria [16,19,20] and medium-chain acyl-CoA dehydrogenase deficiency [16,21,22]. However, most of these studies applied sampling algorithms or reduced data sets, which changes the sick-to-control ratio within the training and test data set compared to NBS [15]. Hence, the applicability of these methods as a daily practice in NBS is unclear, as the ML algorithms are trained and evaluated on these data sets. In this study, we apply ML methods to two data sets, the full data set, containing all screened NBS profiles and the suspected diagnosis data set, where the ML methods are applied in a digital-tier strategy, analogous to a biomarker second-tier, after traditional newborn screening. Overall, we aim to improve NBS for IVA by applying statistical and ML methods on the full and the suspected diagnosis data set. In particular, focusing on the two goals: (1) improved specificity (reduction of false positives) while maintaining 100% sensitivity, and (2) differentiation between mild and classic IVA.

## 2. Materials and Methods

### 2.1. NBS Data Set—Composition, Extraction, and Data Cleaning

About 20% of the newborns in Germany (i.e., about 140,000 newborns per year) are screened at the NBS laboratory at Heidelberg University Hospital (UKHD) [6]. Prior to this study, the UKHD data protection officer checked the set of NBS variables to be anonymized, as well as data extraction and evaluation to be in accordance with the European general data protection regulation (GDPR).

**Figure 1:**
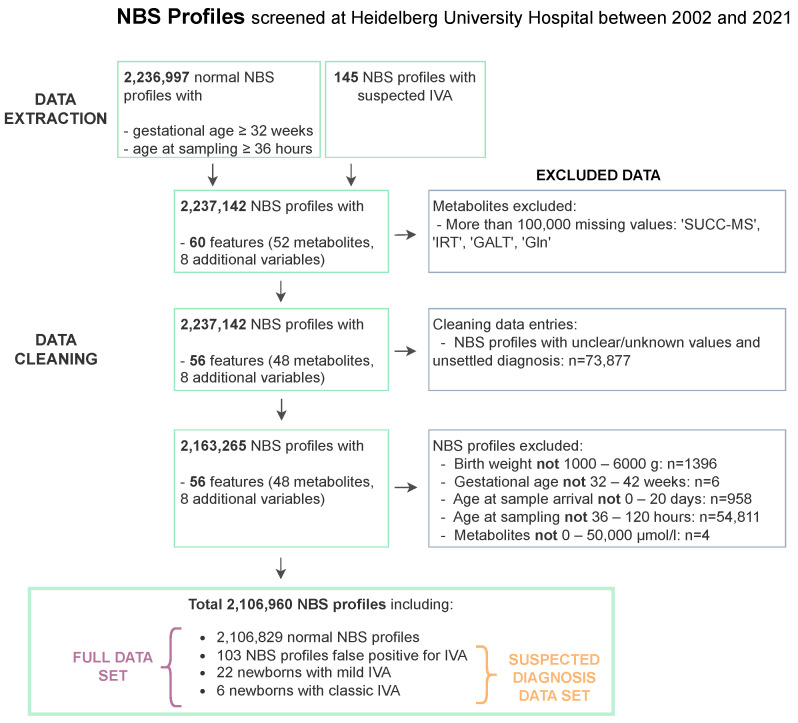
Data extraction and data cleaning flow chart for NBS data screened at the NBS laboratory at Heidelberg University Hospital. NBS profiles from normal and suspected IVA newborns are extracted. From both data sets, features and NBS profiles are excluded due to missing entries and implausible values resulting in 2,106,960 NBS profiles with 60 features including 48 metabolites and 8 additional variables each.

The NBS data set comprises 60 features, which contain 52 metabolites and 8 additional variables that are made up of birth weight, sex, gestational age, birth year, age at blood sample, age at sample arrival, and, if given, the suspected and the subsequently confirmed or excluded diagnosis, Supplementary Table S1A. Figure 1 sums up the data extraction and data cleaning steps performed on the data. For the data extraction, the data set was restricted to first screenings of newborns of at least 32 weeks of gestation, at least 36 hours of life age at sampling, and unremarkable NBS reports (hereafter called ’normal’) to assess the regular NBS, Figure 1. Additionally, all profiles of newborns with suspected IVA, subsequently confirmed (mild/classic IVA) or excluded (false positives) were extracted to the suspected diagnosis data set, Figure 1. Initially, the NBS data set comprised NBS profiles of 2,237,142 newborns (including 145 cases with suspected IVA) born between 2002 and 2021.

Data cleaning of the extracted data set was performed to ensure high data quality and to remove artifacts within the data, Figure 1. First, the metabolite concentrations of glutamine (Gln), succinylacetone (SUCC-MS), immune reactive trypsin (IRT), and galactose-1-phosphate uridyltransferase (GALT) were removed as they were not measured continuously within the time frame and each of them had more than 100,000 missing values. Second, NBS profiles with missing or not interpretable entries such as ‘?’, ‘ ’, ‘U’ were removed. In this step, newborns with an unsettled diagnosis at the time of data extraction were excluded from the data set, which led to a disproportionate exclusion of 8 NBS profiles from the suspected IVA data set. Third, the following ranges were defined to exclude data sets with implausible values: Birth weight: 1000–6000 g; gestational age: 32–42 weeks, age at sampling: 36–120 h, age at sample arrival: 0–20 days and metabolite concentrations: 0–50,000 μmol/L. The categorical variable sex was decoded as ‘0’ for female and ‘1’ for male newborns. Finally, the total data set for analysis (hereafter “full data set”) contained 2,106,090 NBS profiles (including 131 cases with the suspected diagnosis IVA, hereafter: “suspected diagnosis data set”). The suspected diagnosis data set included 28 subsequently confirmed IVA cases (6 classic, 22 mild) and 103 confirmed false positives.

### 2.2. Statistical Methods

In NBS, statistical methods are utilized to analyze the high dimensional data set and find patterns within the complex relationships of metabolites to remove redundant features to improve the accuracy of an algorithm and reduce its training time [23]. Principal component analysis (PCA) reduces dimensionality by focusing on features with the most variation and ranks the principal components by importance, accounting for the most variation in the data [24]. Linear discriminant analysis (LDA) is a supervised method that is applied to maximize the separability between groups. This can be done by projecting the data onto a new axis to simultaneously maximize the distance between the class means and minimize the variation within the classes [24]. T-distributed stochastic neighbor embedding (TSNE) is a method that converts a high-dimensional data set into a matrix of pairwise similarities and visualizes the data similarity [25]. It is capable of capturing the local and global structure of high-dimensional data by revealing the presence of clusters at several scales [25]. Analysis of variance (ANOVA) is a statistical method applied to test if the means of two or more groups are significantly different from each other and therefore examines the impact of one or more factors, which can be used for feature selection [24].

### 2.3. ML Classification

In this section, the ML classification methods, experimental setup, feature sets, and validation procedure are described.

#### 2.3.1. ML Methods

Recently, ML methods have been analyzed in the context of NBS, and from these methods, LR and SVM achieved high performance for NBS classification tasks in comparative studies [15]. LR is a discriminative method that models the posterior probability distribution P(Y|X) of the target variable *Y* given the features *X*. It constructs a separating hyperplane between the classes and for every sample *i* uses the function

$$P\left(z=1|X=x_{i}\right)=\frac{1}{1+e^{-(x_{i}^{T}\beta )}}$$

to consider the distance from the hyperplane as a probability measure of class membership of feature vector $x_{i}\in R^{k}$ with the vector of regression coefficients $\beta =(\beta_{0},\beta_{1},\ldots ,\beta_{k})$. During training, the regression coefficients are fitted using a maximum log-likelihood method to maximize the probability of obtaining the observed results given the fitted coefficients [16,26,27]. Ridge logistic regression (RR) extends this method and has been successfully applied in NBS [22]. It introduces a regularization parameter to the cost function to prevent overfitting, by adding an additional ${\lambda ∥\beta ∥}^{2}$ to the log-likelihood method [28]. We used λ=1.0 as the regularization parameter. SVM attempt to find the largest separating hyperplane between two classes by maximizing the margin between samples and decision boundary [16,27]. A kernel function $K\left(\cdot ,\cdot \right):R^{n}\times R^{n}\to R$ is used for the decision function

$$f\left(x\right)=\sum_{i=1}^{L}\alpha_{i}y_{i}K\left(x_{i},x\right)+b,$$

where $x_{i}\in R^{n}$ are the *L* support vectors which are the nearest training samples to the decision boundary determined from training data, and $y_{i}\in \left\{0,1\right\}$ is the class indicator associated with each $x_{i}$, *b* the bias and $\alpha_{i}\geq 0$ the Lagrange multipliers [16,27]. We used a linear function as the kernel function, and the regularization parameter *c* which controls the trade-off between complexity and allowed classification error was set to 1.0.

**Figure 2:**
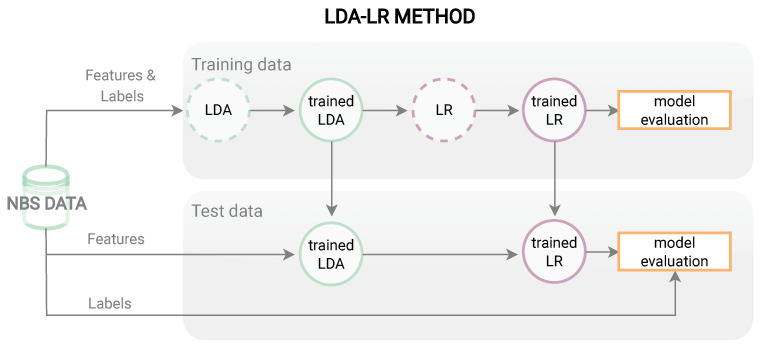
Workflow for LDA-LR method. Showing the training process with features and labels of the training data and evaluation of the trained methods on the test data.

For NBS, it turns out that adapted ML methods, such as using newly constructed features or combining several methods are beneficial [15,21,22,29]. Furthermore, we propose three new combined methods LR based on LDA dimensions (LDA-LR), RR based on LDA dimensions, (LDA-RR) and SVM based on LDA dimensions for application in NBS for IVA. The workflow of these methods is exemplarily presented in Figure 2 for the LDA-LR method. First, the NBS data are split into training and test data. Then the training data including features and labels are used to train an LDA and transform the features to LDA dimensions which are utilized as input to train and subsequently evaluate an LR classifier. The trained LDA and LR models are then applied to the features of the test data set and the predictions are compared to the test data labels to evaluate the model.

#### 2.3.2. Experimental Setup

The experimental setup describes how algorithms were developed and optimized. For all experiments, we used the programming language python (Python Software Foundation. Python Language Reference, version 3.9.2. available at http://www.python.org (accessed on 1 February 2022)). To achieve the overall goal of improving NBS for IVA, each algorithm was applied to the full and suspected diagnosis data set. The latter simulates the scenario, where the ML method is used as an additional step after traditional NBS, a digital-tier, to distinguish false-positive screening results from true positives. Both data sets are subject to data imbalance, where the true positives are in the minority. To overcome this data imbalance, we adapted the class weight parameter $w_{1}$ in each of the classification methods, to penalize a miss-classification of a true positive stronger in the cost function of the optimization step [30]. We used a grid search to find the optimal minority class weight parameter $w_{1}$, while setting the majority class weight parameter $w_{0}=1$ for each method. The algorithms are evaluated on the two objectives, maintaining 100% sensitivity $S_{n}$ and maximizing specificity $S_{p}$,

$$S_{n}=\frac{\mathrm{TP}}{\mathrm{TP}+\mathrm{FN}},\;\mathrm{and}\;S_{p}=\frac{\mathrm{TN}}{\mathrm{TN}+\mathrm{FP}},$$

with true negatives TN, false positives FP, false negatives FN and true positives TP. Hence, the grid search results were first filtered to those maintaining 100% sensitivity, and then the class weight parameter $w_{1}$ was chosen, achieving the results with the highest specificity.

#### 2.3.3. Feature Sets

For ML classifications on the full data set, the two target classes, termed “normal” and “IVA” were applied, where normal included normal NBS profiles and NBS profiles which were false positives for IVA and IVA included newborns with mild and classic IVA as the ML method should classify the normal NBS profiles and newborns with IVA correctly without knowledge of the suspected diagnosis. On the suspected diagnosis data set, the two target classes were termed “normal” and “IVA”, where the label normal was given to the false positives and the label IVA to newborns with mild and classic IVA, as the ML method is supposed to learn to classify the previously false positively diagnosed NBS profiles as normal.

All input features used in the experimental setup were normalized between 0 and 1 to allow for direct comparability of the features. After consultation with clinical experts, we decided to exclude birth year as a feature, as it is highly correlated with the increasing use of pivmecillinam [9] leading to an increase of false-positive screening results, but should not influence the algorithm’s classification. Furthermore, the suspected diagnosis was excluded and the confirmation diagnosis is used as the target variable resulting in 53 features. In recent studies, feature selection techniques were used to improve ML classification for NBS diseases as they can prevent overfitting, and allow classification algorithms to operate faster and more efficiently [15,31]. Hence, besides using all features for the experiments, iteratively adding all significant features according to ANOVA to the feature set, starting with one feature, was evaluated to reduce the feature set to the most meaningful features.

#### 2.3.4. Validation

For evaluation, both data sets were randomly split into 80% training and 20% test set, where the NBS profiles with IVA were split 23 to 5 in training and test set. The classification performance on both data sets was evaluated with the confusion matrix *C*,

$$C=\left[\begin{array}{cc} TN & FP\\ FN & TP \end{array}\right].$$

These results were then validated with ten repeats of 5-fold cross-validation [22]. For the suspected diagnosis data set, the specificity is reported as combined specificity using ML as an additional step to traditional NBS for comparability with the results on the full data set.

## 3. Results

### 3.1. Data Analysis of NBS Data

In NBS, C5 is the primary biomarker to identify newborns with IVA and is until now known to be the best metabolite to discriminate mild and classic IVA in larger cohorts [4,5]. In Table 1, the mean and standard deviation of all 48 metabolites included in the analysis are presented for all normal and false positive NBS profiles as well as newborns with mild and classic IVA. The mean of the measured C5 concentration is highest in newborns with classic IVA (12.6 ± 5.22 μmol/L) and lowest in normal NBS profiles (0.1 ± 0.07 μmol/L) but very similar in newborns with mild IVA (2.6 ± 1.16 μmol/L) and false positive NBS profiles for IVA (2.6 ± 2.06 μmol/L).

For further analysis of the groups of newborns, we applied ANOVA on the full and the suspected diagnosis data sets and evaluated the significant features with a *p*-value p<0.05, Table 2. ANOVA was applied to the full data set with two target classes, normal and IVA, and a data set with three classes, normal, mild IVA, and classic IVA, where normal included the false positives, Table 2a. For the suspected diagnosis data set, ANOVA was applied with two target classes, false positive and IVA, and with three classes, false positive, mild IVA, and classic IVA, Table 2b. C5 is the significant feature with the highest F values on the full NBS data set, Table 2a, and on the suspected diagnosis data set for ANOVA with three target classes, Table 2b. However, tryptophan (Trp) is ranked highest or second highest for the suspected diagnosis data set, whereas it is listed on a lower rank in all methods for the full data set, Supplementary Table S2A. Furthermore, the group of false positive screened newborns has the highest mean value of Trp (102.8 ± 34.42μmol/L) from all groups, and the box plots for Trp show higher concentration values for normal and false positive NBS profiles than for newborns with IVA, Supplementary Figure S1. For all ANOVA evaluations, birth year was identified as a significant feature. The box plots for the birth year show that there are more false positively diagnosed newborns since 2016, Supplementary Figure S1. After consultation with clinical experts, we decided to exclude birth year as a feature for the ML methods, as it is highly correlated with the increasing use of pivmecillinam [9] leading to an increase of false-positive screening results, but should not influence the algorithm’s classification.

**Figure 3:**
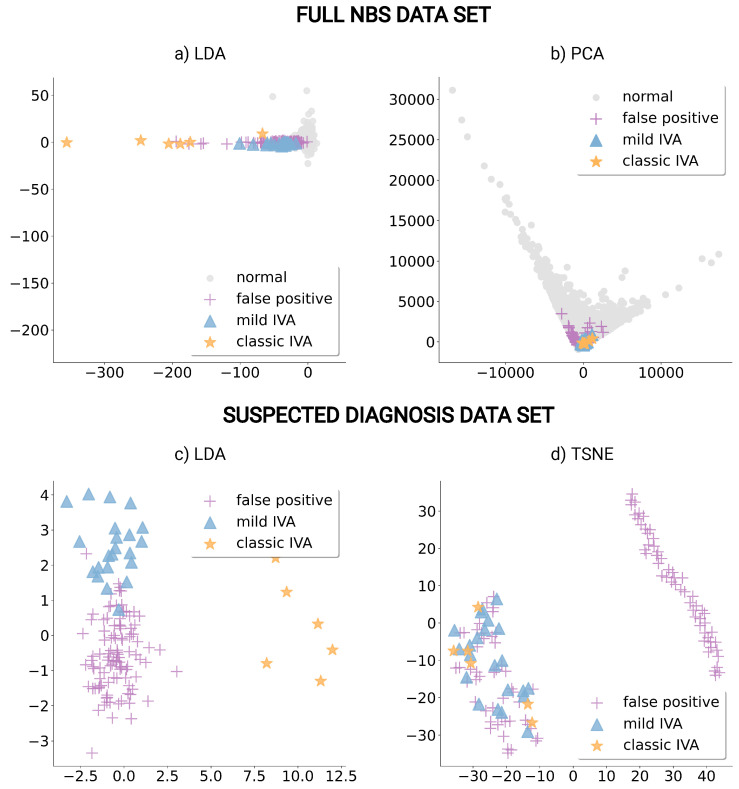
Dimensionality reduction plots of NBS profiles presenting normal in gray (circle) and false positive newborns in purple (cross) as well as newborns with mild IVA in blue (triangle) and with classic IVA in orange (star). Dimensions from linear discriminant analysis (LDA) are presented for the full NBS data set (**a**) and the suspected diagnosis data set (**c**). The first two principal components from principal component analysis (PCA) from the full data set (**b**) and the first two dimensions of T-distributed stochastic neighbor embedding (TSNE) created from the suspected diagnosis data set (**d**) are presented.

For further analysis of the high-dimensional feature space, the dimensionality reduction techniques LDA, PCA, and TSNE were applied to construct meaningful dimensions. These methods revealed clusters of newborns with mild and classic IVA within the reduced dimensions and are presented in Figure 3.

For the full NBS data set, a strict separation of clusters is not possible, although, for both, LDA and PCA, the newborns with mild and classic IVA were more closely grouped with each other and with the falsely positive diagnosed newborns than with the normal NBS profiles, Figure 3a,b. The suspected diagnosis data set transformed into LDA dimensions show three distinct clusters for groups of false positives, mild IVA, and classic IVA, Figure 3c. TSNE for the suspected diagnosis data set separates a large group of false positives from the remaining data, Figure 3d. Hence, for further investigation, we excluded the 5 significant features, based on ANOVA, Trp, argininosuccinate (Asa), 3-methylglutarylcarnitine (MeGlut), 3-OH-tetradecanoylcarnitine (C14OH), and histidine (His) and applied the TSNE algorithm again, the results of this evaluation showed no distinct separation into two clusters, Supplementary Figure S3. Hence, these features seem to have an important influence on the TSNE method. Furthermore, we compared the scatter plots of TSNE dimensions colored depending on the confirmed diagnosis and the classification of an LR algorithm, Supplementary Figure S2. This comparison showed that the LR method identifies all NBS profiles, which can be visually separated from other NBS profiles in TSNE dimensions (right cluster), correctly as normal (purple), Supplementary Figure S2b.

### 3.2. ML Classification Results for IVA Prediction

Based on the presented experimental setup, Section 2.3.1, we trained and optimized the LR, RR, SVM, LDA-LR, LDA-RR, and LDA-SVM classification algorithms to improve NBS for IVA on both data sets and present the classification results in Table 3. For the evaluation of the reduced feature set with ANOVA, we compared false negatives and false positives in the training and test set of each algorithm using significant features, where we chose five features as a trade-off between best classification performance and the number of selected ANOVA features, since adding more features only slightly improved the results, Supplementary Table S3A. The LDA-LR, LDA-RR, and LDA-SVM methods were only applied to the suspected diagnosis data set, as no distinct cluster could be detected in the LDA dimensions for the full data set, Figure 3.

In general, although the optimization procedure described in the experimental set-up aims at maintaining 100% sensitivity, which means 0 false negatives, some methods fail to achieve this in the training and test sets. For the full data set, only LR maintains 100% sensitivity, meaning that all newborns with IVA would be detected. Applying LR on all 53 features reduces the total amount of false positives to 92, whereas only using 5 significant features, increases the total false positives to 209. On the suspected diagnosis data set, all methods maintain 100% sensitivity in the training and test set and reduce the total amount of false positives. In particular, the proposed methods LDA-LR, LDA-RR, and LDA-SVM show a reduction in both training and test sets to cumulative 19, 34, and 22 false positives.

For validation of the presented algorithms, Table 4 presents the mean results of ten repeats of 5-fold cross-validation for all algorithms from Table 3 which maintained 0 false negatives in the training and test sets and improved specificity. For the full data set, LR applied to all features obtained 100% sensitivity and 99.9958% specificity reducing the false positives in total to 88, Table 4. On the suspected diagnosis data set, LDA-RR and LDA-LR obtained the best results, reducing the false positives in total to 31 and 39 while maintaining 100% sensitivity, Table 4. Whereas using LDA dimensions as input features for SVM, and training LR and RR with all 53 features did not maintain 100% sensitivity in the cross-validation. However, the evaluation on the reduced feature set maintained 100% sensitivity for all three algorithms and reduction to 45–47 false positives, which is a reduction of the false positive rate of 54–56% compared to 103 false positives in traditional NBS. Moreover, only using Trp as input for an RR already reduces the false positives to 63, while maintaining 100% sensitivity, which indicates the importance of Trp for the reduction of false positives, Supplementary Table S3A. The results in Table 4 highlight the importance of validating the algorithms, as the initial splitting into training and test set can have a large influence on the performance of an algorithm.

**Figure 4:**
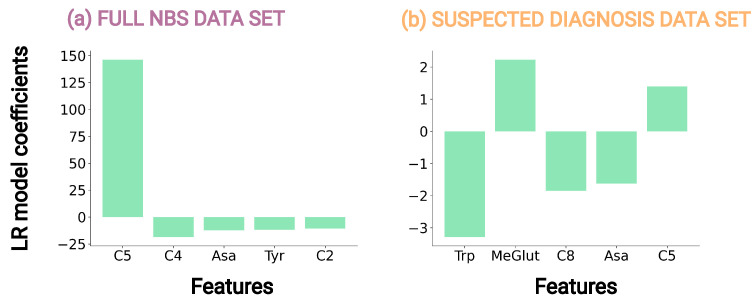
LR model coefficients graph, showing the five highest absolute LR coefficients of the LR models on (**a**) full data set and (**b**) suspected diagnosis data set on all features.

The application of LR allows for an interpretation of its feature contributions, as it is interpretable on a modular level, meaning that it can be inherently explained how parts of the model affect predictions [32]. Hence, we analyzed the LR coefficients $\beta =(\beta_{0},\beta_{1},\ldots ,\beta_{k})$ of the LR models on full and suspected diagnosis data set with all features, Table 3, for interpretation of the methods and analysis of influential metabolite concentrations, Figure 4. Traditional NBS only considers C5, whereas analyzing the coefficients of LR on the full data set identified C5, butyrylcarnitine (C4), Asa, tyrosine (Tyr), acetylcarnitine (C2) as the features with the absolute largest model coefficients, Figure 4a. The coefficient of C5 shows a large positive value, 146, which can be interpreted as an increase in C5 can increase the probability that the NBS profile is classified as a newborn with IVA by the LR model, whereas the increase of a feature value with a negative coefficient such as C4 influences the model in the opposite direction, Supplementary Table S3B. For the suspected diagnosis data set, Trp, octanoylcarnitine (C8), and Asa have the largest negative LR coefficients, whereas MeGlut and C5 have the largest positive LR coefficients, Figure 4a. Trp having the largest negative value, −3.28 can be interpreted as an increase in the feature value of Trp increases the probability that the NBS profile is classified as a normal newborn by the LR model, Supplementary Table S3C. For both data sets, C5 shows large positive coefficients and hence, the interpretation corresponds to an increased C5 level being a known primary marker of the common NBS for IVA [4,5,9].

### 3.3. ML for Newborns with Mild and Classic IVA

The inclusion of IVA into the NBS disease panel resulted in the distinction of individuals with mild and classic IVA [4,5]. Therefore, we analyzed whether ML methods could be applied to classify NBS profiles into three groups, normal, mild IVA, and classic IVA, where normal included normal and false positives for the full data set and only false positives for the suspected diagnosis data set. For this, we chose the best-performing methods from the ML classification, with respect to the highest specificity while maintaining 100% sensitivity in cross-validation, Table 3 and Table 4. The performance of the algorithms was evaluated with a mean confusion matrix of 100 independent runs comparing the predicted and confirmed diagnosis on both data sets.

**Figure 5:**
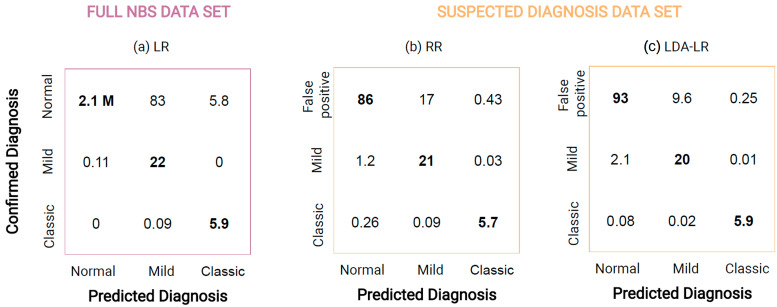
Confusion matrix displaying mean results of 100 independent runs for classification of normal (including false positives), mild IVA, and classic IVA applying (**a**) LR on full NBS data set and for classification of false positive, mild IVA, and classic IVA applying (**b**) RR on suspected diagnosis data set and (**c**) LDA-LR on suspected diagnosis data set. Prediction agreeing with the confirmed diagnosis is highlighted in **bold**.

The mean confusion matrix of LR on the full data set, Figure 5a, shows that the method on average predicts 5.9 of 6 newborns with classic IVA and all newborns with mild correctly while it falsely predicts 88.8 normal NBS profiles as mild IVA and 5.8 normal NBS profiles as classic IVA. On the suspected diagnosis data set, RR reduced the false positives to 17.43, while newborns with mild IVA (21 of 22) and newborns with classic IVA (5.7 of 6) are similar, Figure 5b. Applying the proposed LDA-LR method, the number of false positive newborns reduces to 9.85, while classifying newborns with mild and classic IVA similarly well, Figure 5c.

## 4. Discussion

NBS for IVA in Germany is hampered by the identification of an attenuated disease variant in about 80% of all newborns with confirmed IVA [4], for which the benefit of NBS is still unclear, and an increasing number of false positive screening results due to the use of pivmecillinam contained in the medication. [8]. This study examined the application of data analysis and ML methods as a potential digital-tier on NBS profiles to improve the specificity of NBS for IVA and support the classification of disease severity.

### 4.1. Data Analysis Can Reveal Patterns within NBS Data

The NBS data set from the Heidelberg NBS laboratory contains more than 2 million NBS profiles with 60 features, making the analysis challenging for humans but highly applicable for ML methods, which was already shown for several NBS conditions [15]. The application of ANOVA on the data sets confirmed biological knowledge such as highlighting C5, the known primary variable of the common NBS for IVA [4,5,9] as a significant feature for the full NBS data set, Table 2. Moreover, on both data sets, ANOVA identified birth year as a significant feature, Table 2, for all evaluations, which is explained by the increasing number of false positives in recent years, Supplementary Figure S1, due to the use of pivmecillinam as an antibiotic in pregnant women since its authorization in Germany in March 2016 [8,33]. Other parameters were up to now not described to be altered in newborns with IVA nor are easy to explain regarding the metabolic pathways [4,5,9]. However, for the suspected diagnosis data set, the metabolite concentration of Trp is identified as a significant feature by ANOVA, Table 2, and the LR coefficients, Figure 4, with the false positive newborns having a significantly higher Trp concentration than the newborns with IVA, Table 1. Furthermore, only using Trp as input for RR already reduced the false positives from 103 to 63, Supplementary Table S3A. These results show that Trp plays an important role in the ML methods to improve the classification within the suspected diagnosis data set and hence, in the reduction of false positives. The biochemical explanation for this observation is difficult. A previous study showed that the increased intake of leucine, and by this possibly also its accumulation in IVA, influences intracellular Trp metabolism in rats as they compete for the same amino acid transporters [34].

In NBS, techniques such as PCA have been used to improve NBS for congenital adrenal hyperplasia [29]. For IVA, the methods LDA, PCA, and TSNE showed clusters of newborns with mild and classic IVA within the new dimensions. In particular, for the suspected diagnosis data set, the groups (false positive, mild IVA, classic IVA) could be very clearly separated with LDA dimensions, Figure 3c, indicating the existence of underlying patterns within the data sets that enable a separation of the three newborn groups. The application of the unsupervised method TSNE showed that a large group of false positives could be separated from the remaining data points, Figure 3d, and that this separation was strongly influenced by the significant features, Supplementary Figure S3. Hence, the TSNE method can reveal interesting patterns within the data which the ML algorithm might rely on to reduce the false positives in IVA classification and the ML method can identify features that strongly influence the results of the TSNE method. Application of these methods could also be used to find unknown patterns within large data sets of other NBS conditions analyzing them independently from the data labels.

### 4.2. ML Methods Can Improve IVA Classification

The aim of applying ML methods for IVA classification is to model complex relationships within the NBS data and improve the specificity while maintaining 100% sensitivity. However, this can be a difficult task due to the high data imbalance resulting from the low prevalence of IVA [4,6,7,15]. Most recent studies on ML-based NBS apply sampling algorithms or reduced data sets to overcome data imbalance [16,19,21,29]. however, these methods change the sick-to-control ratio within the training and test data set the ML algorithm is learning and evaluated on, which makes the results difficult to compare to traditional NBS [15]. Hence, in this study, we did not apply these techniques and showed that a grid search over the class weight parameter within the ML cost function for every individual ML algorithm can be applied to overcome the data imbalance problem. Furthermore, current standards in NBS for IVA using C5 as a primary marker are well developed and obtain 100% sensitivity while only suspecting 103 of 2,106,960 (0.05‰) NBS profiles falsely as newborns with IVA. Hence, we evaluated the ML algorithms on two data sets, the full data set, with no prior knowledge, and the suspected diagnosis data set simulating the scenario, where the ML classifier is used as a digital-tier after traditional NBS. Our results showed that the methods improved NBS for IVA more in the latter case, on the suspected diagnosis data set, and especially, the proposed LDA-LR and LDA-RR methods reduced the false positives to 19 and 34 in the training and test set and the false positive rate by 62.1% and 69.9% in cross-validation, Table 3 and Table 4. These results and the improvement of classification, when applying only five significant features, Table 3, highlight and confirm the importance of data analysis and careful feature selection to improve the ML classification for NBS, as shown in previous studies [16,19,21]. Altogether, this suggests that adding ML methods as digital-tier to traditional NBS, similar to the implementation of a biomarker-based two- or multiple-tier strategy, automatized performed in a few minutes if the metabolic first-tier is above the cut-off, could improve NBS for IVA. The increased specificity, i.e., reduction of false positives, could massively reduce harm to the infants and their families with false positive results if further confirmatory diagnostics are not necessary. Furthermore, using ML methods as a digital-tier can support the cost-effectiveness of the IVA NBS. False positive NBS results are accompanied by additional costs and effort, as these results entail the information transmission about the suspicious NBS result to the local hospital and the families by a physician, clinical evaluation of the newborn and sampling for the confirmatory diagnostics by a pediatrician, costs for these metabolic (and genetic) analyses, and finally communication of the results to the families. By reducing the false positive rate by nearly 70% with the digital-tier approach, also these human and material resources could be reduced, while hard- and software costs for the digital-tier approach are low. Moreover, the reduction of the false positives will allow re-focusing the screening for IVA on the true positives.

Since the inclusion of IVA into the NBS disease panel, there has been a distinction between individuals with mild and classic IVA [4,5]. ML methods can classify newborns with mild and classic for both data sets while obtaining a similar false positive reduction, Figure 5. For both data sets, the algorithms used several features as input and false positives were mainly newborns falsely classified as newborns with mild IVA. Until now, isolated C5 is known to be the best metabolite to discriminate mild and classic IVA in larger cohorts [4,5] and may be seconded by other metabolites if a metabolic comprehensive explanation of the combinations is given. As many of the screened individuals with IVA are asymptomatic at the positive screening result [4], it would be crucial to predict the severity of the clinical phenotype in order to enable a stratified diagnostic and therapeutic practice. This would allow immediate treatment of all individuals at risk, and would also reduce over-treatment for individuals with the predominantly identified attenuated variant.

### 4.3. Limitations

The acquired data set consists of more than 2 million NBS profiles but only 28 (22 mild, 6 classic) newborns with IVA. This is above the minimum number of 20 true positives suggested by Lin et al [19] to achieve stable results in NBS but still very low and could negatively affect the proposed ML algorithms, as the true positives might not span throughout the whole feature space needed to be learned by the algorithm. Validating the ML methods with cross-validation, revealed that not all algorithms could robustly obtain good results, indicating that the training and test data sets might not contain sufficient data. Also, the classification results of the three-fold classification, normal, mild, and classic IVA, Figure 5, showed that not all newborns with mild and classic IVA could be identified correctly over the 100 independent runs, which would be an essential requirement for the application of ML methods as a daily practice in NBS. Furthermore, this study only considers data from the Heidelberg screening laboratory in Germany, hence the algorithms could perform differently on other data sets. Moreover, the LDA-LR and LDA-RR methods do not allow for an evaluation of important features since the LR coefficients are only evaluated on the two LDA dimensions. This gives rise to the question if algorithms should only be evaluated based on their performance or also on their interpretability. In general, it is not clear how black box methods can be applied in the clinical context and which ethical and legal requirements are needed to apply ML methods, as well as how these methods may be controlled and accepted by the patients and society [35]. A previous survey in Germany [36] showed that patient representatives expect advances in personalized treatment through the application of AI. They hope for improved interconnected medical care, advanced diagnostics, and the use of electronic patient records. However, strong concerns about data protection and informal self-determination of the data were mentioned. Moreover, the patient representatives fear possible mistakes or discrimination by the algorithms and AI systems due to insufficient training data sets, and the loss of social interaction with physicians [36]. All these concerns should be considered when applying AI methods in the medical context.

### 4.4. Future Directions for ML in NBS

This study is a retrospective study, however, it could also be a direction for future research and prospective future application of ML in NBS. Therefore, the next steps should be to assess the reliability and robustness of the proposed methods on larger data sets, validate the digital-tier strategy in daily practice in parallel to the traditional NBS, and evaluate the possible cost-effectiveness. Thus, a joint analysis of data from different NBS centers would be highly desirable to fulfill the needed positive sample sizes. Furthermore, the proposed ML methods could be applied to other NBS diseases where they could be used to improve specificity but also to enhance the understanding of underlying patterns, and guide directions for future research in the field of biomarker detection. In this study, we focused on ML methods, which were identified in the literature as good candidates for an application to NBS [15]. However, also more advanced methods such as neural networks and adaptive gradient boosting were already applied in NBS [19,37] and could be evaluated for IVA. Moreover, feature attribution and explainable AI techniques could enhance the understanding and interpretability of black box ML methods, which could lead to a higher acceptance of these. Metabolite concentrations, such as Trp, which were highlighted by the algorithms could be investigated in future studies, applying metabolic and model-based approaches such as genome-scale metabolic models, e.g., Recon models [38], which could allow further insights into these alterations. As a last step, the legal and ethical preconditions have to be set up by the responsible authorities to enable ML methods as diagnostic tools.

## 5. Conclusions

In recent years, AI methods, which are trained to learn complex relationships within data, are successfully applied to various tasks within the medical domain [10,11,12]. In this study, we have demonstrated that ML methods can be applied to improve NBS for IVA leading to a reduction of false positives by nearly 70% in cross-validation in a digital-tier strategy and enable increased insight into NBS data. Hence, their nearly cost-less application could be highly beneficial for NBS programs by avoidance of harm to newborns with IVA and their families, less effort for the NBS laboratories in reporting and tracking, and reduction of human and material resources for the confirmatory diagnostics. Furthermore, it opens new perspectives in future NBS research.

## Figures and Tables

**Table 1:** Mean values and standard deviation of 48 metabolite concentrations measured in dried blood samples (μmol/L) for the groups of normal and false positive NBS profiles as well as newborns with mild and classic IVA. The abbreviations can be found in Supplementary Table S1A.

Metabolites	Normal	False Positive	Mild IVA	Classic IVA
	(μmol/L)	(μmol/L)	(μmol/L)	(μmol/L)
NBS Profiles (No.)	2,105,959	103	22	6
17p	9.2 ± 7.22	8.0 ± 4.2	8.8 ± 4.67	14.9 ± 8.72
TSH	2.6 ± 1.75	2.7 ± 1.86	2.9 ± 2.09	0.7 ± 0.47
BIO	0.4 ± 0.09	0.5 ± 0.1	0.5 ± 0.06	0.5 ± 0.09
3HMG	0.0 ± 0.02	0.0 ± 0.01	0.0 ± 0.01	0.0 ± 0.01
Ala	237.1 ± 204.09	268.7 ± 194.71	236.6 ± 116.16	282.5 ± 86.74
Arg	14.7 ± 9.02	14.8 ± 10.97	15.5 ± 6.95	21.2 ± 11.1
Asa	0.6 ± 1.16	2.0 ± 2.34	0.3 ± 0.07	0.2 ± 0.08
Asp	52.8 ± 23.23	67.4 ± 60.31	55.7 ± 17.91	58.5 ± 12.63
C0	21.9 ± 11.35	20.8 ± 15.98	21.1 ± 8.49	24.5 ± 12.39
C10	0.1 ± 0.05	0.1 ± 0.06	0.1 ± 0.06	0.1 ± 0.03
C10:1	0.1 ± 0.05	0.1 ± 0.06	0.1 ± 0.07	0.1 ± 0.02
C12	0.1 ± 0.06	0.1 ± 0.04	0.1 ± 0.08	0.1 ± 0.08
C14	0.2 ± 0.08	0.2 ± 0.09	0.3 ± 0.11	0.3 ± 0.06
C14:1	0.1 ± 0.06	0.1 ± 0.05	0.2 ± 0.09	0.1 ± 0.02
C14OH	0.1 ± 0.03	0.0 ± 0.03	0.1 ± 0.06	0.1 ± 0.04
C16	3.4 ± 1.59	3.3 ± 1.66	3.3 ± 1.8	5.4 ± 1.97
C16:1	0.2 ± 0.08	0.1 ± 0.1	0.2 ± 0.07	0.2 ± 0.04
C16:1OH	0.1 ± 0.02	0.0 ± 0.03	0.1 ± 0.04	0.1 ± 0.08
C16OH	0.0 ± 0.02	0.0 ± 0.03	0.0 ± 0.03	0.0 ± 0.01
C18	1.0 ± 0.33	0.9 ± 0.3	1.0 ± 0.36	1.3 ± 0.37
C18:1	1.1 ± 0.6	1.2 ± 0.7	1.0 ± 0.61	2.0 ± 0.99
C18:1OH	0.0 ± 0.03	0.0 ± 0.03	0.0 ± 0.02	0.1 ± 0.07
C18:2	0.1 ± 0.1	0.2 ± 0.1	0.1 ± 0.06	0.3 ± 0.26
C18OH	0.0 ± 0.02	0.0 ± 0.01	0.0 ± 0.01	0.0 ± 0.01
C2	26.4 ± 12.72	22.8 ± 12.53	24.1 ± 9.56	30.5 ± 11.97
C3	2.1 ± 1.1	2.1 ± 1.3	2.0 ± 0.94	2.6 ± 1.12
C4	0.2 ± 0.14	0.3 ± 0.33	0.2 ± 0.13	0.3 ± 0.13
C5	0.1 ± 0.07	2.6 ± 2.06	2.6 ± 1.16	12.6 ± 5.22
C5:1	0.0 ± 0.02	0.0 ± 0.02	0.0 ± 0.02	0.0 ± 0.02
C6	0.1 ± 0.04	0.1 ± 0.04	0.1 ± 0.03	0.0 ± 0.01
C8	0.1 ± 0.05	0.1 ± 0.06	0.1 ± 0.02	0.1 ± 0.05
C8:1	0.1 ± 0.07	0.1 ± 0.07	0.1 ± 0.08	0.1 ± 0.04
Cit	12.0 ± 6.59	15.0 ± 9.18	12.0 ± 2.99	17.5 ± 12.3
Glu	411.8 ± 103.84	411.8 ± 156.43	415.6 ± 99.99	372.7 ± 63.81
Glut	0.1 ± 0.08	0.1 ± 0.08	0.1 ± 0.09	0.0 ± 0.01
Gly	474.8 ± 166.87	468.8 ± 293.0	494.5 ± 181.09	386.8 ± 68.31
Hci	1.8 ± 1.05	2.0 ± 2.78	1.8 ± 0.58	2.7 ± 2.85
His	448.0 ± 392.35	952.2 ± 770.84	346.7 ± 170.6	230.0 ± 77.2
Leu+Ile	137.8 ± 47.4	145.6 ± 70.45	148.8 ± 62.9	210.3 ± 73.74
MeGlut	0.1 ± 0.04	0.0 ± 0.04	0.1 ± 0.04	0.0 ± 0.01
Met	17.5 ± 7.96	20.6 ± 9.28	17.0 ± 10.84	23.0 ± 8.54
Orn	76.0 ± 78.38	83.5 ± 70.38	75.6 ± 74.08	33.7 ± 63.66
Phe	46.6 ± 13.06	55.5 ± 15.5	45.5 ± 14.01	66.3 ± 29.08
Pro	904.4 ± 440.26	681.6 ± 713.35	985.1 ± 360.82	1203.8 ± 505.32
Thr	119.4 ± 61.79	89.9 ± 109.52	124.0 ± 43.31	125.2 ± 24.0
Trp	78.5 ± 236.31	102.8 ± 34.42	63.6 ± 13.89	51.7 ± 9.78
Tyr	81.3 ± 37.95	96.8 ± 40.64	70.8 ± 34.75	170.2 ± 147.11
Val	102.5 ± 43.83	115.0 ± 64.51	111.3 ± 57.15	194.3 ± 87.61

**Table 2:** ANOVA results with all presented features having a *p*-value p<0.05. All methods were applied to the full NBS data set (a) and the suspected diagnosis data set (b). For 2 class ANOVA, five features with the largest F values with binary target variable normal or IVA, and for 3 class ANOVA, five features with the largest F values with target variable normal (false positive for (b)), mild IVA, and classic IVA are presented. Abbreviations: ANOVA—analysis of variance, C14OH—3-OH-tetradecanoylcarnitine, C16:1OH—3-OH hexadecenoylcarnitine, C18:1OH—3-OH octadecenoylcarnitine, C5—isovalerylcarnitine, His—histidine, MeGlut—3-methylglutarylcarnitine, Trp—tryptophan, Tyr—tyrosine, Val—valine.

2 Class ANOVA		3 Class ANOVA	
(a) Full NBS data set			
Feature	F value	Feature	F value
C5	97,909.21	C5	90,027.83
C16:1OH	28.17	C16:1OH	35.23
age at blood sample	15.74	Tyr	17.29
Val	10.28	Val	13.61
birth year	8.43	C18:1OH	12.90
(b) Suspected diagnosis data set			
Trp	38.86	C5	57.75
birth year	24.37	Trp	19.72
C14OH	22.55	MeGlut	17.00
MeGlut	20.64	birth year	13.30
His	18.22	C16:1OH	13.06

**Table 3:** Evaluation results of ML methods on (a) the full data set and (b) the suspected diagnosis data set. Methods are applied to all 53 features, 5 features selected with ANOVA, or LDA dimensions. The methods were evaluated by false negatives (FN) and false positives (FP) on the training and test set. Abbreviations: Asa—argininosuccinate, aas—age at sampling, BIO—biotinidase, C14OH—3-OH-tetradecanoylcarnitine, C16:1OH—3-OH hexadecenoylcarnitine, C5—isovalerylcarnitine, FN—false negatives, FP—false positives, His—histidine, LDA—linear discriminant analysis, LR—logistic regression, MeGlut—3-methylglutarylcarnitine, RR—ridge logistic regression, SVM—support vector machine, Trp—tryptophan, Val—valine.

Method	Features (Number)	Train FN	Train FP	Test FN	Test FP
(a) Full data set					
LR	all (53)	0	65	0	27
RR	all(53)	6	20,065	3	5005
SVM	all(53)	1	35	0	9
LR	C5, C16:1OH, aas, Val, BIO	0	167	0	42
RR	C5, C16:1OH, aas, Val, BIO	5	6026	1	1577
SVM	C5, C16:1OH, aas, Val, BIO	1	68	0	15
(b) Suspected diagnosis data set					
LR	all (53)	0	29	0	7
RR	all (53)	0	18	0	5
SVM	all (53)	0	20	0	6
LR	Trp, C14OH, MeGlut, His, Asa	0	35	0	6
RR	Trp, C14OH, MeGlut, His, Asa	0	35	0	6
SVM	Trp, C14OH, MeGlut, His, Asa	0	37	0	6
LDA-LR	LDA dimensions	0	9	0	10
LDA-RR	LDA dimensions	0	22	0	12
LDA-SVM	LDA dimensions	0	12	0	10

**Table 4:** Mean results of ten repeats of 5-fold cross-validation on (b) the full data set and (c) suspected diagnosis data set, compared to traditional screening results (a). Methods are applied to all 53 features, 5 features selected with ANOVA or LDA dimensions. The methods were evaluated by sensitivity, specificity, and number of false positives (real numbers are rounded up) combined for training and test set. For the suspected diagnosis data set, these evaluations are calculated based on the full data set to allow comparability between both data sets. Abbreviations: Asa—argininosuccinate, C14:1—tetradecenoylcarnitine, C14OH—3-OH-tetradecanoylcarnitine, C5—isovalerylcarnitine, FN—false negatives, FP—false positives, His—histidine, LDA—linear discriminant analysis, LR—logistic regression, MeGlut—3-methylglutarylcarnitine, RR—ridge logistic regression, SVM—support vector machine, Trp—tryptophan.

Method	Features (Number)	Sensitivity % (FN)	Specificity %	FP
(a) Traditional NBS				
NBS	C5	100	99.9951	103
(b) Full data set				
LR	all (53)	100	99.9958	88
(c) Suspected diagnosis data set				
LR	all (53)	98.2143 (1)	99.9981	41
RR	all (53)	96.2143 (2)	99.9987	29
SVM	all (51)	97.9286 (1)	99.9975	52
LR	Trp, C14OH, MeGlut, His, Asa	100	99.9978	47
RR	Trp, C14OH, MeGlut, His, Asa	100	99.9979	46
SVM	Trp, C14OH, MeGlut, His, Asa	100	99.9979	45
LDA-LR	LDA dimensions	100	99.9981	39
LDA-RR	LDA dimensions	100	99.9985	31
LDA-SVM	LDA dimensions	99.2857 (1)	99.9985	32

## Data Availability

The data are not publicly available due to privacy restrictions.

## Supplementary Materials

The following supporting information can be downloaded at: https://www.mdpi.com/article/10.3390/metabo13020304/s1, Figure S1: Box plots for feature tryptophan (Trp) on full data set and suspected diagnosis data set and box plots for birth year and C5; Figure S2: Feature selection overview; Figure S3: Machine learning (ML) classification results; Table S1: Data overview; Table S2: Feature selection overview; Table S3: Machine learning (ML) classification results.

## Abbreviations

The following abbreviations are used in this manuscript:

AI	artificial intelligence
ANOVA	analysis of variance
Asa	argininosuccinate
C2	acetylcarnitine
C4	butyrylcarnitine
C5	isovalerylcarnitine
C8	octanoylcarnitine
GALT	galactose-1-phosphate uridyltransferase
GDPR	general data protection regulation
Gln	glutamine
His	histidine
IRT	immune reactive trypsin
IVA	isovaleric aciduria
LDA	linear discriminant analysis
LR	logistic regression
MeGlut	3-methylglutarylcarnitine
ML	machine learning
NBS	newborn screening
PCA	principal component analysis
RR	logistic ridge regression
SUCC-MS	succinylacetone
SVM	support vector machine
Trp	tryptophan
TSNE	t-distributed stochastic neighbor embedding
Tyr	tyrosine
UKHD	Heidelberg University Hospital
