# Supplementary material for: Machine Learning Methods Improve Specificity in Newborn Screening for Isovaleric Aciduria
Source: Metabolites. 2023 Feb 18;13(2):304. doi: 10.3390/metabo13020304 (PMC9962193; doi:10.3390/metabo13020304)

Supplemental Materials: Machine learning methods improve specificity in newborn screening for isovaleric aciduria

1. **Supplementary Table 1:** Data overview
    - **A:** Overview of all metabolite concentrations and additional variables screened at the NBS laboratory at Heidelberg University Hospital.
2. **Supplementary Table 2:** Feature selection overview
    - **A:** ANOVA on full data set (p values and F values) for every feature.
    - **B:** ANOVA on suspected diagnosis data set (p values and F values) for every feature.
3. **Supplementary Table 3:** Machine learning (ML) classification results
    - **A:** ML classification results as confusion matrix for training and test set for full and suspected data sets.
    - **B:** Logistic regression (LR) coefficients for LR classification on full data set applied on all features.
    - **C:** LR coefficients for LR classification on suspected diagnosis data set applied on all features.
4. **Supplementary Figure 1:** Box plots for feature tryptophan (Trp) on full data set and suspected diagnosis data set and box plots for birth year and C5.
5. **Supplementary Figure 2:** Scatter plots of TSNE dimensions on suspected diagnosis data set colored according to confirmed diagnosis and ML prediction.
6. **Supplementary Figure 3:** Scatter plot of TSNE dimensions on suspected diagnosis data set excluding 5 features identified by ANOVA feature selection procedure.

Supplementary Figures

Figure S1: Box plot of NBS data for Trp on full data set and suspected diagnosis data set and box plots of birth year and C5 for the groups normal, false positive (FP), mild IVA and classic IVA.

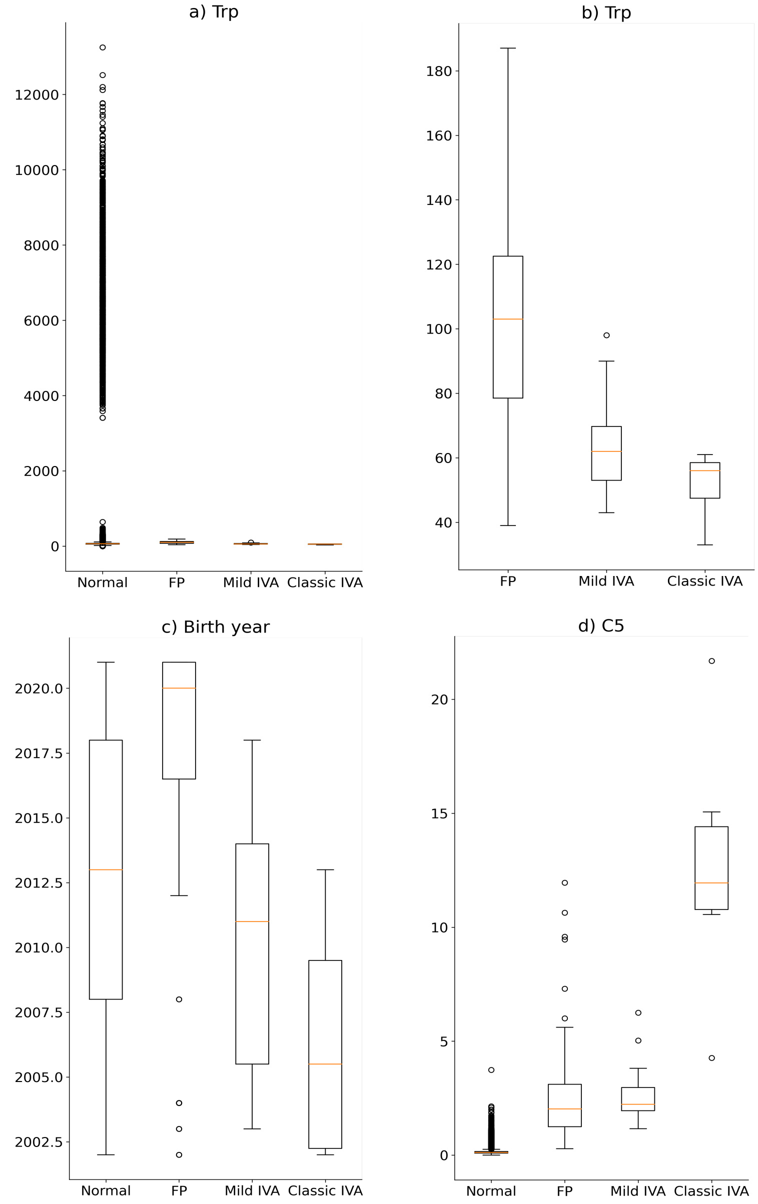

Figure S2: TSNE dimensions of suspected diagnosis data set presented with (a) color mapping depending on confirmed diagnosis and (b) color mapping depend-ing on predictions by logistic regression (LR) method. Purple points represent newborns classified as normal by LR and green points represent newborns clas-sified as newborns with IVA by LR. All false positive newborns (gray) that can be visually separated from the remaining newborns in TSNE dimensions (a) are correctly classified as normal newborns (purple) by the LR method (b).

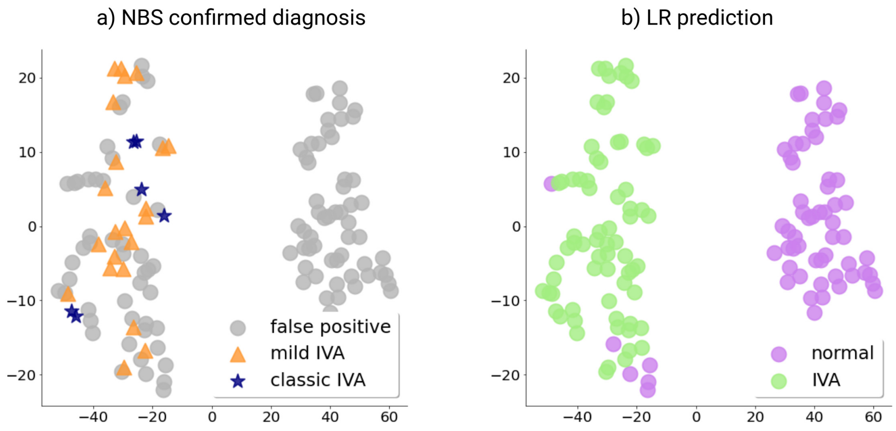

Figure S3: TSNE dimensions of suspected diagnosis data excluding the features Trp, Asa, MeGlut, C14OH and His, which were significant features according to analysis of variance (ANOVA) and used for the improved ML classification. No distinct separation of groups in the TSNE dimensions can be identified visually.

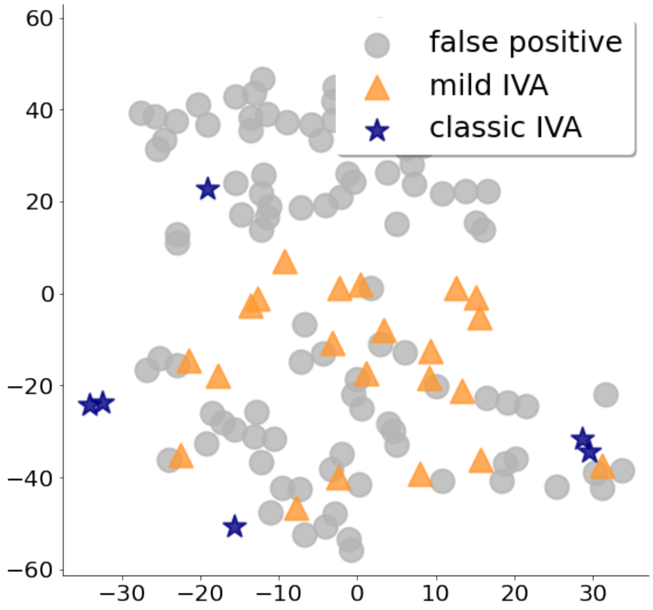

Supplement: Supplementary file 1 [file metabolites-13-00304-s001.zip › Supp_material.pdf]
